# Extraction of uranyl from spent nuclear fuel wastewater via complexation—a local vibrational mode study

**DOI:** 10.1007/s00894-024-06000-4

**Published:** 2024-06-18

**Authors:** Bárbara M. T. C. Peluzo, Renaldo T. Moura Jr., Elfi Kraka

**Affiliations:** 1 Computational and Theoretical Chemistry Group (CATCO), Department of Chemistry, Southern Methodist University, 3215 Daniel Avenue, Dallas, TX 75275–0314 USA; 2 Department of Chemistry and Physics, Center of Agrarian Sciences, Federal University of Paraíba, Areia, 58397–000 Paraíba Brazil

**Keywords:** Spent nuclear fuel wastewater, Uranium extraction, Uranyl, Uranyl amide complexes, Uranyl Schiff base complexes, Local vibrational mode analysis

## Abstract

**Context:**

The efficient extraction of uranyl from spent nuclear fuel wastewater for subsequent reprocessing and reuse is an essential effort toward minimization of long-lived radioactive waste. N-substituted amides and Schiff base ligands are propitious candidates, where extraction occurs via complexation with the uranyl moiety. In this study, we extensively probed chemical bonding in various uranyl complexes, utilizing the local vibrational modes theory alongside QTAIM and NBO analyses. We focused on (i) the assessment of the equatorial O-U and N-U bonding, including the question of chelation, and (ii) how the strength of the axial U$=$O bonds of the uranyl moiety changes upon complexation. Our results reveal that the strength of the equatorial uranium-ligand interactions correlates with their covalent character and with charge donation from O and N lone pairs into the vacant uranium orbitals. We also found an inverse relationship between the covalent character of the equatorial ligand bonds and the strength of the axial uranium-oxygen bond. In summary, our study provides valuable data for a strategic modulation of N-substituted amide and Schiff base ligands towards the maximization of uranyl extraction.

**Method:**

Quantum chemistry calculations were performed under the PBE0 level of theory, paired with the relativistic NESCau Hamiltonian, currently implemented in Cologne2020 (interfaced with Gaussian16). Wave functions were expanded in the cc-pwCVTZ-X2C basis set for uranium and Dunning’s cc-pVTZ for the remaining atoms. For the bonding properties, we utilized the package LModeA in the local modes analyses, AIMALL in the QTAIM calculations, and NBO 7.0 for the NBO analyses.

**Graphical abstract:**

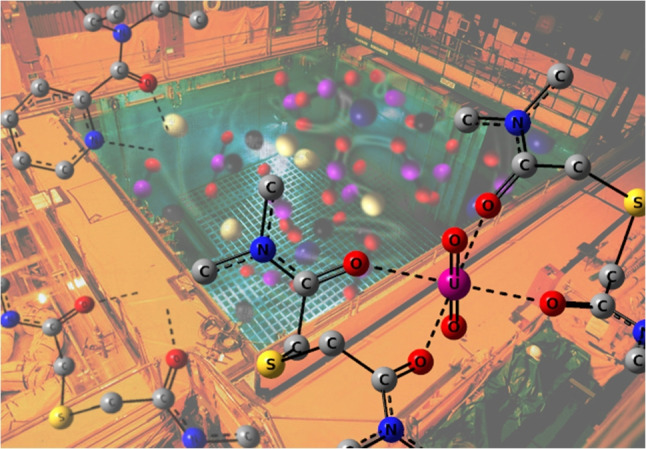

**Supplementary Information:**

The online version contains supplementary material available at 10.1007/s00894-024-06000-4.

## Introduction

Nuclear energy protects air quality by efficiently producing massive amounts of carbon-free electricity [1, 2]. In 2022, power plants generated more than 2500 billion kWh worldwide accounting for around 60% of energy production in countries, such as France and Slovakia, and supplying communities in more than 28 U.S. states with electric power [3]. However, malfunctions of nuclear power plants can be catastrophic, as documented by the Chernobyl [4] and the Fukushima disasters [5]. Furthermore, nuclear reactors generate the so-called spent nuclear fuel (SNF) with highly radioactive waste [6] that has to be managed according to the International Atomic Energy Agency safety standards [1, 3]. After being discharged from the reactor, SNF is first kept in storage racks, which are placed in a water pool, serving as a cooling medium as well as radiation protection before direct disposal in deep geological repositories or recycling the reusable and/or burnable components and disposing only the residual waste [6–10].

**Fig. 1:**
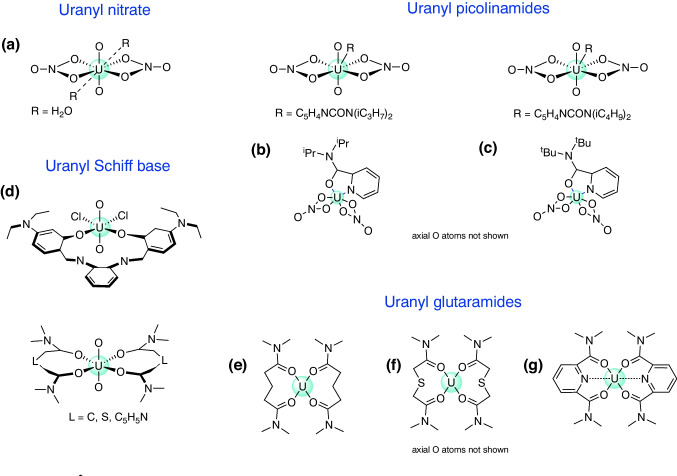
Schematic representation of the seven uranyl complexes investigated in this work: **(a)** uranyl nitrate, **(b)** uranyl-N, N-diisopropyl pyridine-2-carboxamide, **(c)** uranyl-N, N-diisobutyl pyridine-2-carboxamide, **(d)** uranyl-N, N’-bis[(4,4’-diethylamino)salicylidene]-1, 2-phenylenediamine, **(e)** uranyl-diglutaramide, **(f)** uranyl-dithiodiglycolamide, **(g)** uranyl tetraalkylpyridine-2, 6-dicarboxamide

Because uranium is mainly used in the nuclear fuel cycle [6], the main component of the radioactive SNF wastewater is uranium in the form of the UO$_2^{2+}$ dication, called uranyl. It can easily penetrate the soil, harming animals and plants, because it interferes with numerous biochemical processes [11, 12], e.g., reducing white blood cells and/or leading to different forms of cancer [13, 14].

For decades, chemists and engineers have explored different ways to extract UO$_2^{2+}$ from the radioactive wastewater for reprocessing. Current extraction technologies include chelating agents [10, 15, 16], separation by membranes [17], sorbent chemistry [18, 19], adsorption on metal-organic frameworks (MOFs) [20], or carbon nanotubes [21]. The complexation of UO$_2^{2+}$ with proteins has been suggested because proteins offer more than one binding site capturing UO$_2^{2+}$ [22–24].

Nitrates, N-substituted amides, and Schiff bases have been reported as promising uranyl extractants [10, 25]. Some representatives are shown in Fig. 1. They are fully combustible, show high hydrolytic and radiation stability, and produce innocent degradation products [26–28]. N-substituted amides show a diverse coordination behavior, being prone to binding through either the carbonyl oxygen atom or the deprotonated N, or even both offering the chelation possibility, i.e., leading to a strengthening of metal-ligand bonding via bidentate coordination. In particular, picolinamide-based ligands have shown prominent extraction capabilities and high selectiveness towards actinides/lanthanides [29–33] as observed by Das et al. [34] for N,N-diisopropylpyridine-2-carboxamide and N,N-diisobutylpyridine-2-carboxamide, which bind the uranyl nitrate moiety via 5-membered chelates, see Fig. 1, complexes **(b)** and **(c)**. Diamides, such as glutaramide, are bidentate ligands that coordinate the uranyl moiety place via their carbonyl oxygens [35], see uranyl-diglutaramide **(e)**. It has been suggested that extraction can be enhanced by the replacement of the central glutamaride carbon with sulfur, see uranyl-dithioglutaramide **(f)**, which alters the ligand electronic structure [36]. Alternatively, diamides derived from dipicolinic acid, such as pyridine-2,6-dicarboxamide, have been suggested as potential tridentate ligands with the pyridine N atom being prone to bind uranium [36–38]. Schiff bases have received attention due to their ability to complex different metal ions; e.g., Schiff base ligands have been probed as potential uranium extractants from soil and seawater [39]. The N,N’-bis[(4,4’-diethylamino)salicylidene]-1,2-phenylenediamine ligand (see complex **(d)** in Fig. 1) has been previously used by Klamm et al. [40, 41] for the complexation of a range of metals in the IV oxidation state, including Th, U, and Pu. In a subsequent study, the authors utilized the protonated ligand in the synthesis of an uranyl complex, with potential applications in uranium extraction [42].

The stability of the formed uranyl complexes has been addressed via thermodynamics studies of the chelation process [43], binding energies [33, 36], and indirectly via stretching of the uranyl moiety [44]. Although these efforts have provided some valuable insides, the nuclear community is still looking for specific descriptions of individual uranium interactions in those complexes. In particular, detailed knowledge about the individual uranium-ligand bond strengths in these complexes is mandatory for deriving a protocol on how to strategically modulate a specific ligand to maximize uranium extraction. Some attempts have been made in this direction to estimate the uranium-ligand bond strengths in a few uranyl complexes via electronic density [42, 45] or natural bond orbitals approaches [46]. However, these properties are not well suited for a quantitative description of the bond strength, as frequently pointed out in the literature [47–55]. Our local vibrational mode analysis (LMA) [55, 56], originally introduced by Konkoli and Cremer [57, 58], is much better suited for this purpose.

We evaluated in this work the strength of uranium-ligand interactions for the set of seven uranyl complexes shown in Fig. 1, including uranyl nitrate dehydrate [U(NO$_3$)$_2\cdot$2 H$_2$O], Fig. 1, complex **(a)**, which is usually formed in SNF wastewater [59].

The article is organized as follows: The next section provides the theoretical methodology employed as well as a brief introduction of LMA. Then, the obtained results are discussed, presenting the optimized structures of the seven uranyl complexes, followed by a discussion of the strength of the uranyl-ligand interactions in these complexes assessed with LMA complemented with Bader’s QTAIM (quantum atoms in molecules) [60–62] and the NBO (natural bond orbital) analyses [63–65]. Finally, conclusions are presented suggesting the best candidate for a strong uranium-ligand binding.

## Computational details

The most rigorous quantum-mechanical description of a system containing uranium is through the 4-component Dirac equation [66, 67] since relativistic effects are non-negligible in the bottom region of the periodic table. Nonetheless, its practical implementation is computationally demanding for the majority of systems. Several relativistic methods have been derived over the years, attempting to include the main relativistic effects of interest from a chemist’s perspective. One example is Dyall’s NESC method (Dirac-exact NESC) [68], subsequently reformulated by Cremer, Filavoc, and Zou, allowing for accurate calculations of first- and second-order response properties [69–72]. A comprehensive discussion of NESC implementations proposed by Cremer, Filatov, and Zou is given in Ref. 73. Recently, Zou implemented a new variant of the NESC method, including atomic unitary transformations for which the term NESCau/X2Cau was coined [74], allowing for highly accurate relativistic calculations of large systems, such as actinides metallocenes with more than 600 electrons, recently published [75, 76].

To quantitatively access the strength of the U-O and U-N interactions, we utilized LMA. Information on the electronic structure of a molecule, such as the strength of its bonds, is encoded in the normal vibrational modes. However, these modes are generally delocalized caused by a coupling of the atomic movements during the vibration [77–80]. This hinders direct access to this valuable information. LMA provides a unique solution to this problem by extracting local vibrational modes and related local properties from the normal vibrational modes [55, 56]. In this work, we utilized local mode stretching force constants $k^a$, which reflects the intrinsic strength of a chemical bond or weak chemical interaction [81]. Over the past years, we have successfully applied local mode force constants to characterize the strength of covalent bonds and non-covalent interactions across the periodic table [55, 56], including bonding inside the active site of proteins  [82–87]. LMA combined with NESC has been successfully applied to characterize the metal-ligand interactions in lanthanides [88, 89] and actinides [6, 75, 76], including bonding in uranium complexes. Recently, LMA was expanded for solid-state systems [90–92] and used for the description of uranium-based materials [93]. For a detailed description of LMA, we refer the reader to two recent review articles [55, 56].

For the comparison of larger sets of $k^a$ values, the use of a relative bond strength order BSO $\textit{n}$ is more convenient. Both are connected according to the generalized Badger rule derived by Cremer, Kraka, and co-workers [47, 94], via the following power relationship:

1 $$\begin{aligned} BSO ~n = u\left( k^a\right) ^v \end{aligned}$$

**Table 1:** Geometric, vibrational, and electronic data for the U-O$_{ax}$, U-O, U-N, U-S, and U-Cl interactions in complexes **(a)**–**(g)**; UO$_2^{2+}$ and its neutral counterpart UO$_2$ previously investigated by our group [6]; as well as for the reference molecules used to determine BSO *n* (Eqs. 2 and 3)

				*r*	$k^a$	BSO *n*	MBO *n*	*H*(**r**$_b$)	$\Delta E^{(2)}$
(a)	$^1A_g$	$C_{2h}$	U-O$_{ax}$	1.748	7.568	2.019	2.268	$-$2.292	1.07
			U-O$_{NO_3}$	2.458	0.842	0.430	0.498	$-$0.060	10.51
			U-O$_{H_2O}$	2.527	0.646	0.357	0.323	$-$0.013	2.70
(b)	$^1A$	$C_1$	U-O$_{ax}$	1.751	7.453	1.997	2.245	$-$2.257	33.94
				1.756	7.123	1.934	2.211	$-$2.205	41.01
			U-O$_{NO_3}$	2.458	0.820	0.422	0.458	$-$0.061	104.10
				2.468	0.792	0.412	0.490	$-$0.056	98.33
				2.442	0.859	0.436	0.517	$-$0.069	109.08
				2.458	0.816	0.421	0.503	$-$0.059	106.46
			U-O$_{^iPr}$	2.448	0.573	0.328	0.394	$-$0.035	78.83
			U-N	2.735	0.381	0.209	0.323	$-$0.016	49.83
(c)	$^1A$	$C_1$	U-O$_{ax}$	1.750	7.450	1.997	2.243	$-$2.265	37.37
				1.756	6.999	1.911	2.198	$-$2.207	41.64
			U-O$_{NO_3}$	2.457	0.802	0.416	0.464	$-$0.060	102.93
				2.461	0.798	0.414	0.496	$-$0.059	99.73
				2.443	0.857	0.436	0.516	$-$0.068	109.18
				2.455	0.824	0.424	0.490	$-$0.063	106.65
			U-O$_{^tBu}$	2.482	0.561	0.323	0.362	$-$0.037	70.58
			U-N	2.708	0.404	0.220	0.332	$-$0.020	53.41
(d)	$^1A$	$C_1$	U-O$_{ax}$	1.754	7.047	1.920	2.219	$-$2.228	33.45
				1.758	6.858	1.883	2.207	$-$2.184	31.27
			U-O	2.411	0.656	0.361	0.408	$-$0.049	105.42
				2.406	0.616	0.345	0.411	$-$0.051	109.45
			U-Cl	2.639	1.137	−	−	$-$0.113	177.68
				2.633	1.216	−	−	$-$0.115	181.46
			NH$\cdots$O	1.696	0.281	−	−	$-$0.067	−
				1.718	0.263	−	−	$-$0.056	−
(e)	$^1A_g$	$C_{2h}$	U-O$_{ax}$	1.741	7.515	2.009	2.267	$-$2.363	10.74
			U-O	2.449	0.431	0.268	0.459	$-$0.034	6.89
(f)	$^1A$	$D_{2}$	U-O$_{ax}$	1.748	6.662	1.845	2.250	$-$2.293	29.48
			U-O	2.332	0.928	0.461	0.498	$-$0.078	118.0
(g)	$^1A$	$C_{2}$	U-O$_{ax}$	1.742	7.634	2.031	2.253	$-$2.348	43.61
			U-O	2.461	0.565	0.325	0.423	$-$0.032	98.59
				2.462	0.559	0.322	0.418	$-$0.032	99.21
			U-N	2.750	0.573	0.300	0.313	$-$0.011	45.28
UO$_2^{2+}$	$^1\Sigma _g^+$	$D_{\infty h}$	U-O$_{ax}$	1.682	10.637	2.566	2.543	$-$3.721	19.58
UO$_2$ [6]	$^3\Phi _u$	$D_{\infty h}$	U-O$_{ax}$	1.775	7.288	1.966		$-$2.092	
UO$_2$(OH)$_2$	$^1A$	$C_2$	U-O$_{ax}$	1.762	7.114	1.932	2.270	$-$2.162	9.10
			U-O	2.100	2.791	1.0	1.175	$-$0.418	33.6
H$_2$N−UH	$^3A'$	$C_s$	U-N	2.166	2.253	1.0	1.150	$-$0.306	< 0.5 $^a$
HN$=$UH$_2$	$^5A$	$C_1$	U$=$N	1.872	4.865	1.97	2.265	$-$0.298	< 0.5 $^a$

Bond distances *r* are given in Å; the associated local force constants $k^{a}$ in mdyn/Å; *H*(**r**$_b$) is given in Ha/Å$^3$; the 2$^{nd}$ order stabilization energy $\Delta E^{(2)}$ accounts for charge transfers from the O, N, S, and Cl lone pairs into vacant uranium orbitals, and it is given in kcal/mol. In addition to BSO *n*, also Mayer bond orders (MBO *n*) are given. NESCau/PBE0//cc-pwCVTZ-X2C(U)/cc-pVTZ model chemistry

$^a$ Result below the threshold for printing in the NBO 7.0 code (0.5 kcal/mol)

The constants *u* and *v* are calculated from $k^a$ values of two reference compounds with known BSO $\textit{n}$ values and the requirement that for a zero force constant the corresponding BSO $\textit{n}$ value is zero. For example, for CC bonds, suitable references are ethane and ethylene with bond orders n $=$ 1 and n $=$ 2, respectively. In the case of metal-ligand bonding [95], we usually refer to Mayer bond orders MBO *n* [96–98]. For the uranyl-oxygen bonds, we chose the U-O and U-O$_{ax}$ bonds of uranyl hydroxide (UO$_2$(OH)$_2$) as reference molecule with MBO *n* of 1.175 and 2.270, respectively, which corresponds to a ratio of 1:1.932. For the uranyl-nitrogen bonds, we used the pair H$_2$N-UH and HN$=$UH$_2$ as reference molecules with MBO *n* of 1.150 and 2.265, respectively, which corresponds to a ratio of 1:1.970. Utilizing the scaled MBO *n* of 1 and 1.932 for uranyl-oxygen bonds and 1 and 1.970 for uranyl-nitrogen bonds (see Table 1), we derived the following power relationships for uranyl-oxygen and uranyl-nitrogen bonds following the protocol described in recent work [6]:

2 $$\begin{aligned} BSO ~n~(U\text {-}O)= &   0.4855\left( k^a\right) ^{0.7041}\end{aligned}$$

3 $$\begin{aligned} BSO ~n~(U\text {-}N)= &   0.4894\left( k^a\right) ^{0.8803} \end{aligned}$$

To assess the covalent character of U-O and U-N bonds, we applied the *Cremer-Kraka criterion* for covalent bonding [99–101], which is composed of two conditions: *Necessary condition.* The existence of a bond critical point **r**$_b$ on the electron density bond path between the two atoms under consideration. *Sufficient condition.* If the energy density *H*(**r**) at **r**$_b$ is negative, the interaction between the two atoms is of covalent character; if *H*(**r**$_b$) is positive, the interaction is predominantly electrostatic. H$( {\textbf {r}})$ is defined as

4 $$\begin{aligned} H(\textbf{r}) = G(\textbf{r}) + V(\textbf{r}) \end{aligned}$$

with the kinetic and potential energy densities *G*(**r**) and *V*(**r**), respectively [60–62].

We investigated the complex stabilization energies via the charge donation from the lone pairs of the ligand atoms (i.e., oxygen, nitrogen, and sulfur) into the empty orbitals of uranium. To do so, we calculated the 2$^{nd}$-order stabilization energy, $\Delta E^{(2)}$, based on 2$^{nd}$-order perturbation theory, where the perturbation is the deviation from the ideal Lewis structure [102]. $\Delta E^{(2)}$ is calculated in the natural bond orbitals (NBO) basis, and it is given by

5 $$\begin{aligned} \Delta E^{(2)}_{i^*} = \frac{\langle \varphi _i^{(0)} | \hat{H} | \varphi _{j^*}^{(0)} \rangle ^2}{\varepsilon _{j*}^{(0)}-\varepsilon _i^{(0)}} \end{aligned}$$

where $\varphi ^{(0)}$ is an unperturbed NBO with energy $\varepsilon ^{(0)}$, and the subscripts *i* and *j* account for occupied and unoccupied orbitals, respectively. $\hat{H}$ is the Hamiltonian used.

For the actual calculations, we utilized the following program packages. Relativistic effects were included through the relativistic Hamiltonian NESCau [74], currently implemented in Cologne2020 [103], interfaced with Gaussian16 [104]. Geometry optimizations and frequency calculations were performed with the PBE0 level of theory [105–107] utilizing the cc-pwCVTZ-X2C basis set for uranium [108] and Dunning’s cc-pVTZ for the remaining atoms [109]. Hybrid functionals have been successfully applied for the description of uranyl systems, including the complexes investigated in this work [36, 42]. It is noteworthy that uranyl diamide complex geometries calculated in the gas-phase tend to be in good agreement with experimental geometries measured in solution [36]. All basis functions were obtained from the Basis Set Exchange Database [110–112]. For LMA, we used the LModeA program package [113]. QTAIM calculations were performed with the AIMALL package [114], and the NBO analysis with NBO 7.0 [115].

## Results

**Fig. 2:**
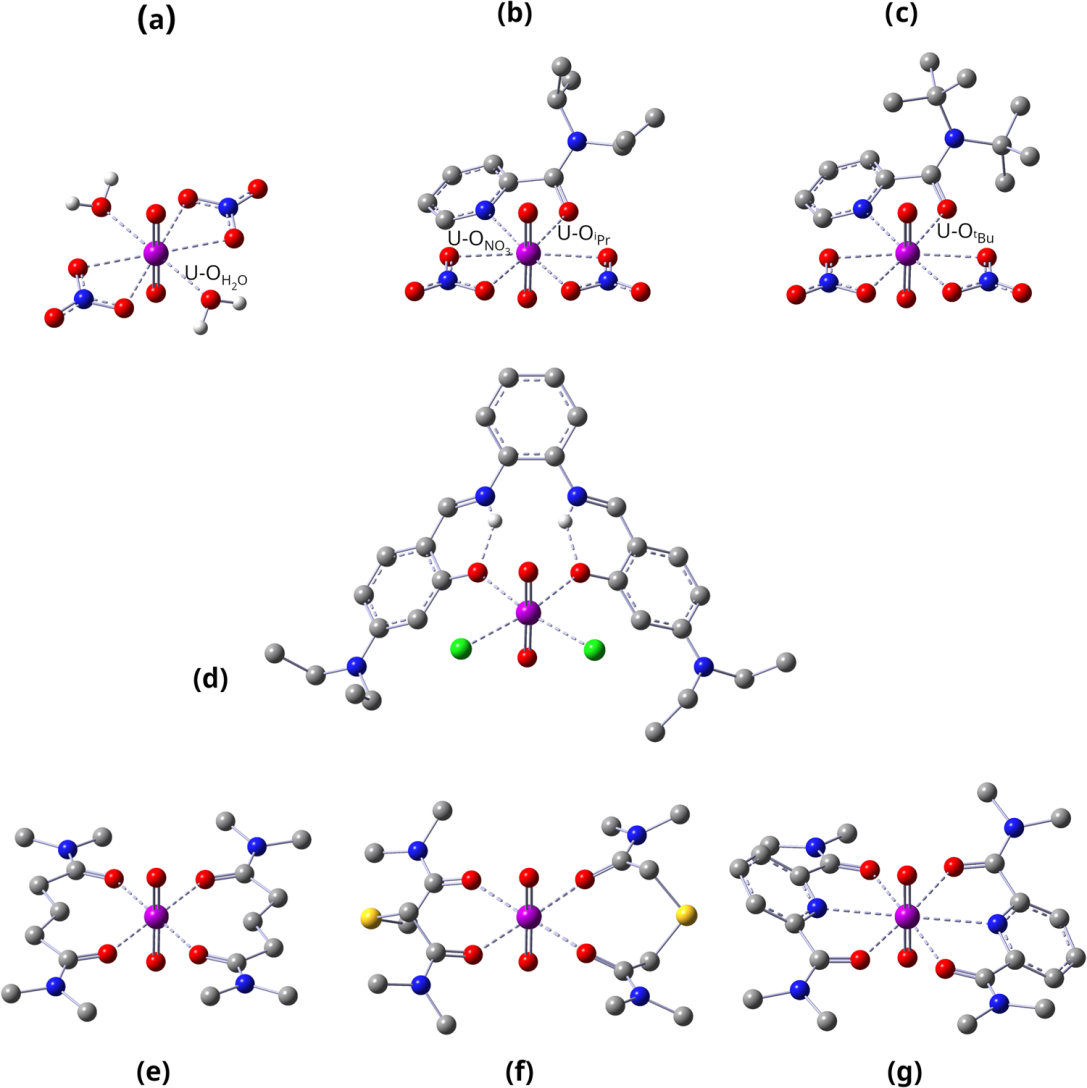
Optimized geometries for all complexes studied. Hydrogens were omitted for clarity, except for **(a)** and **(d)**, where hydrogens from the aquo group and the NH$\cdots$O fragment, respectively, are shown. U-O$_{NO_3}$, U-O$_{H_2O}$, U-O$_{^iPr}$, and U-O$_{^tBu}$ interactions are indicated. Gray: carbon, red: oxygen, blue: nitrogen, green: chlorine, yellow: sulfur, light-gray: hydrogen, purple: uranium. NESCau/PBE0//cc-pwCVTZ-X2C(U)/cc-pVTZ

Table 1 gathers results on all uranium-ligand interactions. Optimized structures are shown in Fig. 2. Throughout the paper, the following notation is utilized. U-O$_{ax}$ refers to U-O bonding in the UO$_2^{2+}$ moiety. U-O refers to bonding between uranium and equatorial oxygen atoms, O$_{NO_3}$ refers to binding between uranium and oxygen atoms of the NO$_3$ units (complexes **(a)**–**(c)**); O$_{H_2O}$ refers to the water oxygens in **(a)**; O$_{^iPr}$ refers to the oxygens of the $^i$Pr substituted picolinamide-based ligand (complex **(b)**); similarly, O$_{^tBu}$ refers to $^t$Bu oxygens (complex **(c)**). U-N refers to U-N binding. The utilized nomenclature is illustrated in Fig. 2.

**Fig. 3:**
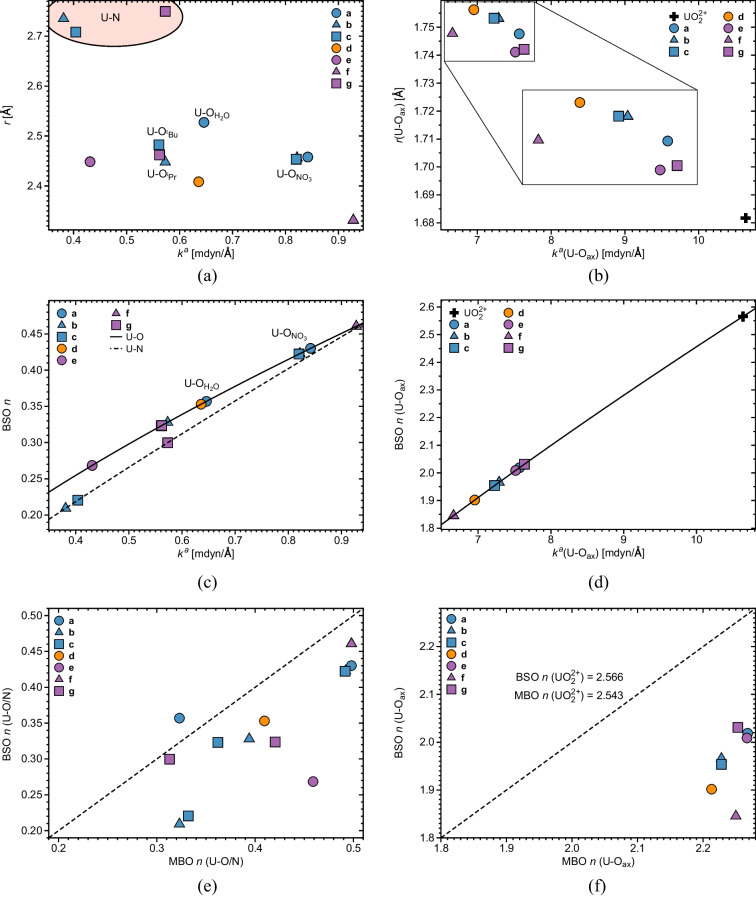
**a** Equatorial interatomic distances *r* vs. local force constants $k^a$ for U-O and U-N interactions. For complexes **(a)**-**(c)**, and **(g)**, nonequivalent U-O$_{H_2O}$, U-O$_{NO_3}$, U-O$_{^iPr}$, and U-O$_{^tBu}$ interactions are indicated, see text and Fig. 2 for explanation. U-N interactions in **(b)**, **(c)**, and **(g)** are highlighted by a pink ellipsis.   **b** Axial interatomic distances *r*(U-O$_{ax}$) vs. local force constants $k^a$ for complexes **(a)**–**(g)** and uranyl. **c** BSO *n* curves for U-O (solid line) and U-N (dashed line) interactions, calculated via Eqs. 2 and 3. For better readability, the plots in Fig. 3 show average values for the U-O$_{NO_3}$ interactions in **(b)** and **(c)**, and U-O interactions in **(d)** and **(f)**. Absolute values are reported in Table 1.   **d** BSO *n* curve for the axial U-O$_{ax}$ bonds, calculated via Eq. 2. **e** BSO *n* versus MBO *n* for the U-O and U-N interactions. **f** BSO *n* versus MBO *n* for the axial U-O$_{ax}$ bonds. The values of MBO *n* BSO *n* for uranyl are written. NESCau/PBE0//cc-pwCVTZ-X2C(U)/cc-pVTZ

**Fig. 4:**
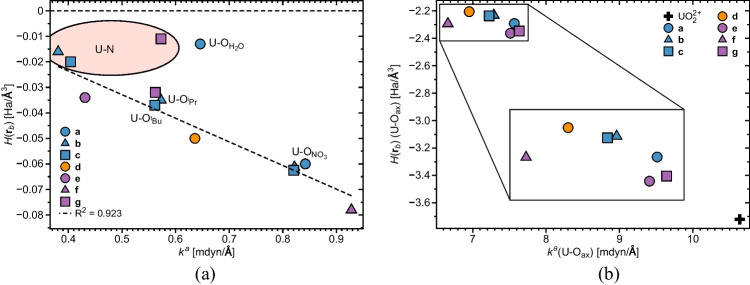
**a** Energy density evaluated at the bond critical point *H*(**r**$_b$) of the equatorial U-O (U-N) interactions vs. their associated local force constant $k^a$. Results pertaining to U-O$_{H_2O}$ interactions in **(a)** and U-N interactions in **(g)** were disregarded in the linear regression. For complexes **(a)**–**(c)** and **(g)**, nonequivalent U-O$_{H_2O}$, U-O$_{NO_3}$, U-O$_{^iPr}$, and U-O$_{^tBu}$ interactions are indicated, see text and Fig. 2 for explanation. For better readability, the plots show average values for the U-O$_{NO_3}$ interactions in **(b)** and **(c)**, and U-O interactions in **(d)** and **(f)**. Absolute values are reported in Table 1. U-N interactions in **(b)**, **(c)**, and **(g)** are highlighted by a pink ellipsis. For an explanation on the bonds notation, see Fig. 2. **b** Energy density evaluated at the bond critical point *H*(**r**$_b$) of the axial U-O$_{ax}$ interactions vs. their associated local force constant $k^a$(U-O$_{ax}$). NESCau/PBE0//cc-pwCVTZ-X2C(U)/cc-pVTZ

**Fig. 5:**
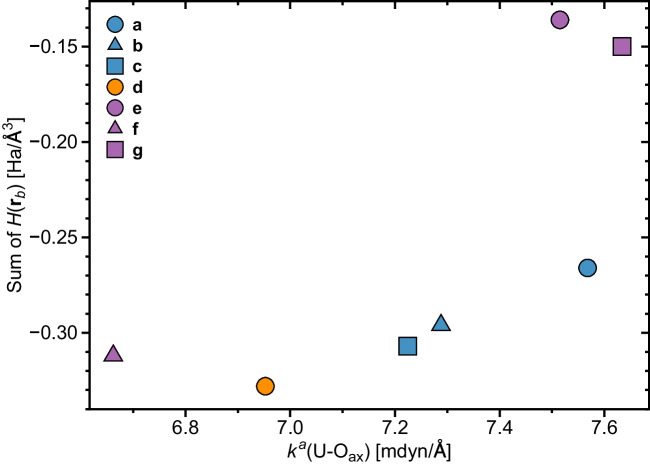
Relationship between the sum of equatorial *H*(**r**$_b$) values taken for all U-ligand bonds and the *H*(**r**$_b$) values of the axial U-O$_{ax}$ bonds. NESCau/PBE0//cc-pwCVTZ-X2C(U)/cc-pVTZ

**Fig. 6:**
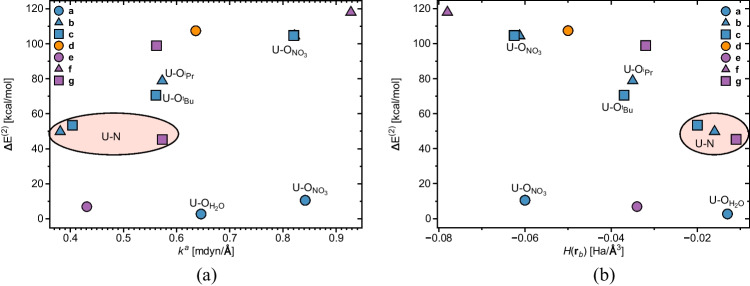
**a** Evolution of $\Delta E^{(2)}$ concerning oxygen (nitrogen) charge donations toward uranium vs. the local force constant associated with U-O (U-N) interactions. **b** Evolution of $\Delta E^{(2)}$ concerning oxygen (nitrogen) charge donations toward uranium as a function of *H*(**r**$_b$) evaluated at the corresponding U-O (U-N) bond critical points. For complexes **(a)**–**(c)** and **(g)**, nonequivalent U-O$_{H_2O}$, U-O$_{NO_3}$, U-O$_{^iPr}$, and U-O$_{^tBu}$ interactions are indicated, see text and Fig. 2 for explanation. For better readability, the plots show average values for the U-O$_{NO_3}$ interactions in **(b)** and **(c)**, and U-O interactions in **(d)** and **(f)**. Absolute values are reported in Table 1. U-N interactions in **(b)**, **(c)**, and **(g)** are highlighted by a pink ellipsis. For an explanation on the bonds notation, see Fig. 2. NESCau/PBE0//cc-pwCVTZ-X2C(U)/cc-pVTZ

**Fig. 7:**
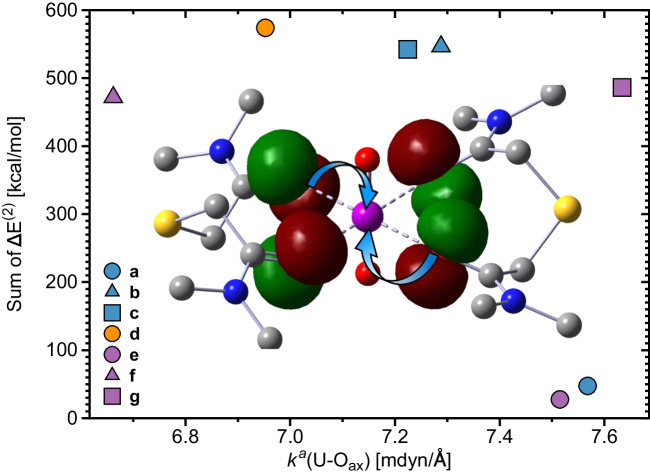
Relationship between the overall $\Delta E^{(2)}$ contributions originating from equatorial interactions and the strength of U-O$_{ax}$ bonds. An example of how the charge donations take place is shown for complex **(f)**. For better readability, we did not show charge donations from all donors. NESCau/PBE0//cc-pwCVTZ-X2C(U)/cc-pVTZ

For better readability, the plots in Figs. 3, 4, 5, 6 and 7 show results averaged over U-O$_{NO_3}$ interactions in **(b)** and **(c)**, U-O in **(d)** and **(g)**, and U-O$_{ax}$ in **(b)**–**(d)**. The use of averaging is justified by minor differences in the results pertaining to those interactions and does not change the overall picture. Individual U-O interactions are reported in Table 1. We first discuss optimized geometries and LMA results, followed by QTAIM and 2$^{nd}$-order stabilization energy data.

### Geometries and LMA

Uranyl nitrate dihydrate **(a)** adopts $C_{2h}$ symmetry with the two water ligands and the two nitrate groups arranged on opposite sites of the equatorial plane, and the nitrate hydrogens out of this plane (see Fig. 2). U-O$_{NO_3}$ and U-O$_{H_2O}$ bond distances are 2.458 and 2.527 Å, respectively. U-O$_{NO_3}$ interactions have local force constants of 0.842 mdyn/Å  and with that being slightly stronger than U-O$_{H_2O}$ (0.646 mdyn/Å) interactions, as can be perceived in Fig. 3a.

In contrast, we observed a lack of symmetry for complexes **(b)**–**(d)** containing bulky ligands, whereas the uranyl glutaramides **(e)**-**(f)** adopt $C_{2h}$, $D_{2}$, and $C_{2}$ symmetry, respectively, as shown in Fig. 2.

All ten complexes studied in this work exhibit small local force constants, in particular when compared to other systems containing uranium, such as uranium metallocenes [76, 91]. Nonetheless, it should be pointed out that the ten complexes studied in this work were previously experimentally characterized [33, 36, 42]. Moreover, smaller $k^a$ values have been observed for other heavy metal chelate systems, such as Eu(III) chelates [89]. Therefore, the magnitude of $k^a$ values does not necessarily imply weak extraction properties. More important is the number of interactions and the fact that the U(VI) atom in uranyl allows for higher coordination.

Figure 3 shows the relationship between equatorial U-O and U-N, and U-O$_{ax}$ bond distances and their corresponding local mode force constants. The majority of the equatorial U-O ligand bonds (see Fig. 3a and Table 1) exhibit bond distances clustering around 2.5 Å, while the corresponding U-N distances are longer, measuring between 2.708 and 2.750 Å. U-O$_{ax}$ bonds in **(a)**–**(g)** cluster around 1.75 Å, i.e., they are considerably longer compared to the U-O bonds of the uranyl moiety with a bond length of 1.682 Å  (see Fig. 3b and Table 1). As revealed in Fig. 3a and b, bond distances and associated local mode force constants qualitatively follow a Badger relationship [116], i.e., shorter bonds are also stronger bonds, which is not always the case, as frequently reported in the literature [48].

U-N ligand bonds are somewhat weaker than the majority of U-O ligand bonds. However, with BSO *n* values in a range between 0.20 and 0.45, they are both considerably weaker than a single bond with a BSO *n* of 1, as revealed by Fig. 3c. Bond distances and associated local mode force constants of U-O$_{ax}$ bonds also qualitatively follow a Badger relationship, as obvious from Fig. 3b. BSO *n* values of all U-O$_{ax}$ bonds are of double bond character with BSO *n* values ranging from 1.85 to 2. The strongest U-O$_{ax}$ bonds are found for uranyl; however, with a BSO *n* value of 2.566, they do not reach triple bond character (see Fig. 3d). This interesting point will be discussed in more detail below.

Complexes **(b)** and **(c)** exhibit similar geometries, where NO$_3$ groups and the picolinamide ligand are oriented in a distorted hexagonal bipyramidal geometry (see Fig. 2). Our molecular structures are consistent with experimental solid-state structures [33]. Introduction of the picolinamide ligands did not alter LMA results pertaining to U-O$_{NO_3}$ interactions, i.e., **(a)**, **(b)**, and **(c)** exhibit nearly similar results concerning the distance and strength associated with U-O$_{NO_3}$ interactions, as can be noticed in Fig. 3a. On the other hand, while U-O$_{^iPr}$ (U-O$_{^tBu}$) and U-O$_{\textrm{NO}_3}$ distances are somewhat comparable in **(b)** and **(c)**, ranging between 2.4 and 2.5 Å, the strength associated with the latter is higher (see Fig. 3a and Table 1), reflecting a change in the electronic environment brought on by the incorporation of the ligand. We did not observe major differences in the strength and distance of U-O$_{^iPr}$ and U-O$_{^tBu}$ interactions (see Fig. 3a), unveiling that the alkyl substituent does not affect U-O interaction strength, similar to Das et al. [33] observations on the same systems. U-N distances are considerably larger than U-O$_{^iPr}$ (U-O$_{^tBu}$), being equal to 2.735 and 2.708 Å  in the $^i$Pr and $^t$Bu substituted complexes, respectively. The pattern of internuclear U-O$_{^iPr}$ (U-O$_{^tBu}$) distances being larger than U-N was previously observed in similar systems [33, 117–119]. We identified non-negligible interactions between uranium and nitrogen with local force constants of 0.381 and 0.404 mdyn/Å  (see Fig. 3a). Such results collect evidence that nitrogen is a potential binding site in **(b)** and **(c)**, revealing the bidentate character of both ligands, regardless of the alkyl substituent, as suggested by Das et al. [33].

Despite the lack of symmetry, U-O and U-N distances in **(d)** are almost equivalent and clustered around 2.41 and 4.56 Å, respectively. The larger U-N distance suggests the absence of interactions between the two atoms. The Schiff base ligand slightly bends in a “soft taco” arrangement, as can be seen in Fig. 2, in line with the solid-state structure characterized by Klamm et al. [42]. According to LMA, U-O interactions in **(d)** have an average value of 0.636 mdyn/Å. The magnitude of such interaction is comparable to the value reported by Moura Jr. et al. [89] in Eu(III) complexes featuring similar ligands. U-Cl bonds have strong interactions, measuring 1.137 and 1.216 mdyn/Å  in agreement with the study conducted by Coupez and Wipff [43], where it was found that uranyl complexes containing U-Cl interactions exhibited higher binding energies than their counterparts featuring NO$_3^-$ ligands. Klamm et al. [42] suggested the existence of intramolecular hydrogen bonds of the type N-H$\cdots$O, i.e., between the hydrogens connected to the nitrogens and binding oxygens (see structure **(d)** in Fig. 2), due to the fact N-H units provoked a bend of the ligand towards oxygen atoms, forming a “pocket” shape. Motivated by this assumption, we have utilized LMA on the assessment of the magnitude of the strength associated with such oxygen-hydrogen interactions. The values obtained, 0.281 and 0.263 mdyn/Å, are comparable to hydrogen bondings of the type N-H$\cdots$O reported elsewhere [120–122], revealing that complex **(d)** is further stabilized by intramolecular hydrogen bondings.

Optimized geometries for **(e)**, **(f)**, and **(g)** exhibit $C_{2h}$, $D_2$, and $C_2$ symmetry, respectively, in agreement with published results [36]. Carbonyl oxygens serve as potential binding sites because they are positioned around the uranium, in line with experimental findings [36]. The glutaramide ligand is arranged in a planar fashion around the uranyl moiety, as can be seen in Fig. 2-complex **(a)**, with a U-O bond distance of 2.449 Å  and corresponding local force constant of 0.431 mdyn/Å  (see Fig. 3a and Table 1), which is in good agreement with the EXAFS distance of Chen and co-workers Chen et al. [36] (2.41 ± 0.02 Å). Furthermore, the local force constants reflect the coordination pattern in solution observed by the same authors. Thiodiglycolamide ligands in complex **(f)** bend off the equatorial plane, and their U-O distance of 2.332 Åis smaller than that of the U-O distance in the glutaramide complex. This goes in line with a pronounced increase in the U-O local force constant (0.928 mdyn/Å, and corresponding BSO *n* of 0.461, still substantially below a single bond), suggesting that the change in the electronic environment caused by sulfur atoms is affecting the actual strength of the U-O interactions. This result is in line with Sasaki and Tachimori [123] observation that U(IV) extraction with thiodiglycolamide ligands is higher than the one provided by other diamide extractants without donor groups, as for the glutaramide ligand. On the other hand, the local force constant (6.662 mdyn/Å)  of the U-O$_{ax}$ bond in **(f)** is smaller than that of **(e)** (7.515 mdyn/Å) although both complexes exhibit similar U-O$_{ax}$ distances, reinforcing the hypothesis that the thiodiglycolamide ligands change the electron density distribution throughout the complex. The large U-S distance of 4.814 Å  indicates the absence of any direct interaction between U and S, confirming previous suggestions [124].

Similarly to complex **(f)**, complex **(g)** exhibits a nonplanar arrangement of the equatorial ligands, as obvious from Fig. 2. The U-O distance in **(g)** is similar to that found for complex **(e)**, although the U-O bond strength in **(g)** is slightly larger; a result that can be associated with the presence of the pyridine group in the ligand, altering the electronic environment. The U-N distance of 2.750 Å in **(g)** is similar to the U-N distances in **(b)** and **(c)** (2.735 and 2.708 Å, respectively). The local force constant of U-N in complex **(g)** is 0.573 mdyn/Å  which corresponds to a BSO *n* of 0.300, a value that is comparable to the BSO *n* values of 0.320 and 0.325 found for U-O bonds in **(g)**. This is an important proof of the hypothesis that the nitrogen lone pair of the pyridine-2,6-dicarboxamide ligand is capable of binding uranium [36, 38, 125, 126], conveying its tridentate ligand character in complex **(g)**. Comparing the U-O$_{ax}$ bond strength in **(e)**, **(f)**, and **(g)**, we find a bond strength order **(f)** < **(e)** < **(g)** with BSO *n* values of 1.845, 2.009, and 2.031. This result clearly reveals that the S atom in the thiodiglycolamide ligand does not only influence the strength of the equatorial U-O bonds in **(f)**, i.e., making them stronger, it also influences the axial U-O$_{ax}$ bonds, i.e., making them weaker. A previously published bond strength order **(e)** < **(f)** < **(g)**, indirectly calculated based on binding energies [36], does not match our results. However, it has to be noted that binding energies are cumulative properties and therefore are not suited as quantitative bond strength measure, as has been discussed in the literature [127]. Moreover, the fact that the ligands in **(f)** have tridentate character, while **(e)** and **(g)** contain bidentate ligands, warns that a direct comparison with binding energies should be made with care.

In Fig. 3e and f, BSO *n* and MBO *n* values are correlated for U-O (U-N) interactions and axial U-O$_{ax}$ bonds, respectively. MBO *n* usually exhibit classical integer values for homonuclear diatomics, whereas non-integer values have been found for in more complex bonding situation including transition metal bonding [128]. As discussed by these authors, in the latter situations, MBO *n* reflects in addition to the intrinsic strength of the bond, the ionic character of the bonds as well as delocalization and multi-center effects, in contrast to our BSO *n* which only accounts for the intrinsic bond strength. As such, MBO *n* values are larger than their BSO *n* counterparts, as clearly demonstrated in Fig. 3e and f. It is interesting to note that for uranyl, both MBO *n* and BSO *n* values are almost identical (2.543 and 2.566, respectively), i.e., delocalization and multi-center effects are small, and as a consequence, both MBO *n* and BSO *n* reflect the intrinsic bond strength. However, in the uranyl-complexes, a different picture emerges. The strength of the axial U-O$_{ax}$ bonds is reduced in response to delocalization of electronic over the complex. As a consequence, MBO *n* accounting for both effects remains fairly constant (values between 2.20 and 2.27), in contrast to the BSO *n* (values between 1.84 and 2.03) reflecting the intrinsic axial U-O$_{ax}$ bond strength. In this way, the difference between MBO *n* and BSO *n* offers the opportunity to disclose delocalization and multi-center effects. Follow up work will focus on this interesting point also comparing both with a recently suggested delocalization index [129].

According to our geometry optimization, UO$_2^{2+}$ is a $^1\Sigma _g^+$ species belonging to the $D_{\infty h}$ point group. Both U-O$_{ax}$ bonds have a distance of 1.682 Å, in line with previously published studies [130, 131]. The associated local U-O$_{ax}$ force constants of 10.637 mdyn/Å  are stronger than those of its neutral counterpart (UO$_2$) in the $^3\Phi _u$ state [6]. The derived BSO *n* for uranyl is 2.566, reflecting a bond strength between double and double-triple bond. According to molecular orbital theory, uranyl formally exhibits triple UO bonds, where the hybridized 5f and 6d orbitals of uranium overlap with oxygen’s 3p, forming $\sigma$ and $\pi$ bonds. Nonetheless, as pointed out by Denning [132], the actual contribution of those orbitals for the overall chemical interaction is not completely clear and therefore molecular orbital theory offers a controversial interpretation of the uranyl bond order. It also has to be mentioned that the NBO analysis conducted by Klamm et al. [42], where the 6 bonding orbitals in uranyl were found to be highly polarized, and an extensive study conducted by Clark et al. [133], where a variety of wave function methods were applied to the description of actinides systems, did result in a broad range of possible bond orders for the uranyl UO bonds. As can be seen from Fig. 3c, the U-O$_{ax}$ distance increases upon complexation, a result that is similar to Pyykko and Zhao [130] observations on uranyl-oxygen clusters. Complexes featuring voluminous ligands, **(b)**–**(d)**, tend to exhibit larger U-O$_{ax}$ bond distances, suggesting that the overall bond length expansion is a result of steric hindrance. The U-O$_{ax}$ bond length increase implies bond weakening. A much debated question is how to explain the U-O$_{ax}$ bond weakening as ligands are added to the uranyl moiety. Tsushima [134], using orbital population analysis, attributed the U-O$_{ax}$ bond weakening to a decrease in bond covalency and a competition between the ligand and O$_{ax}$ orbitals. Di Pietro and Kerridge [135] found a strong correlation between the degree of covalency of equatorial ligand interactions and the strength of U-O$_{ax}$ bonds. Several authors associated U-O$_{ax}$ bond weakening with charge donation from ligand lone pairs into empty uranium orbitals [44, 119, 136, 137]. In the next sections, we utilize QTAIM and NBO bond analysis tools to rationalize the U-O$_{ax}$ bond weakening encountered in the seven complexes investigated in this study.

### QTAIM analysis

According to the Cremer-Kraka criterion [99–101], the uranium-ligand bonds of all complexes studied in this work exhibit covalent character as reflected by slightly negative *H*(**r**$_b$) values ranging from $-$0.08 and $-$0.01 Ha/Å$^3$, see Table 1 and Fig. 4a and b, with a good agreement on previous results on actinides metallocenes [75, 76]. According to our results, U-O bonds are slightly more covalent than U-N bonds in agreement with previous observations in U(IV) complexes [40]. The degree of covalency of the equatorial bonds increases with the corresponding local force constant, except for the U-N bond in **(g)** and the U-O$_{H_2O}$ bonds in **(a)**, with an R$^2$ value of 0.9210 (obtained by excluding the aforementioned outliers). The *H*(**r**$_b$) values of the axial uranium-oxygen bonds, see Table 1 and Fig. 4b, range from $-$2.2 to $-$3.72 Ha/Å$^3$, i.e., they are more covalent than their equatorial U-O counterparts, as they exhibit double bond character [100]. Figure 4b depicts the relationship between *H*(**r**$_b$) (U-O$_{ax}$) and corresponding $k^a$ values. From this plot, one notices a pronounced decrease of the covalent character of the U-O$_{ax}$ bonds as ligands are incorporated into the uranyl moiety, i.e., UO$_2^{2+}$ exhibits the largest covalency ($-$3.721 Ha/Å$^3$), while for complexes **(a)**–**(g)**, *H*(**r**$_b$) values cluster around $-$2.3 Ha/Å$^3$. This results is in line with previous studies on uranyl complexes [42]. Similarly to the results pertaining to the equatorial U-O and U-N bonds, U-O$_{ax}$ bonds follow the qualitative trend that the more covalent bonds are also the stronger bonds, as revealed from Fig. 4b.

U-O$_{NO_3}$ bonds in **(a)** are slightly more covalent than U-O$_{H_2O}$, with respective *H*(**r**$_b$) values of $-$0.06 Ha/Å$^3$ and $-$0.013 Ha/Å$^3$. Incorporation of picolinamide-based ligands does not alter the covalency of the U-O$_{NO_3}$ bonds nor their strength as revealed by the data for complexes **(b)** and **(c)**, see Table 1. On the other hand, U-O$_{^iPr}$ and U-O$_{^tBu}$ bonds in complexes **(b)** and **(c)** are more covalent than the U-O$_{H_2O}$ interactions in **(a)**, as depicted in Table 1. QTAIM calculations identified bond critical points between uranium and nitrogen atoms of the picolinamide-based ligands in **(b)** and **(c)**. With energy density values of $-$0.016 and $-$0.020 Ha/Å$^3$ in **(b)** and **(c)**, respectively, this reinforces the conjecture that the picolinamide-based ligands act as bidentate ligands in uranyl complexes, regardless of the alkyl substituent [33] with the U-N ligand bonds being slightly weaker and less covalent than their U-O$_{^iPr}$ and U-O$_{^tBu}$ counterparts. It should be mentioned that Das et al. [33] observed little covalent contributions, originating from the picolinamide ligands, in the same complex.

The strength and covalency of U-O bonding in complex **(d)** is of intermediate character compared to the other complexes investigated in this work, as revealed from the data in Table 1. No bond critical point was found between nitrogen and uranium, confirming the absence of U-N bonding in **(d)**. Instead, our QTAIM analysis identified hydrogen bonding between the H atom of the NH unit and the neighboring O atom, see Fig. 2 and Table 1, with energy density values of $-$0.056 and $-$0.067 Ha/Å$^3$, reflecting their covalent character. U-Cl bonds exhibit strong covalency reflected by *H*(**r**$_b$) values of $-$0.115 and $-$0.113 Ha/Å$^3$ being about 2.3 times larger in magnitude than the corresponding U-O values. These findings are in line with a study by Klamm et al. [42], based on interacting quantum atoms (IQA) energy decomposition analysis, which suggested that the electrostatic contributions of U-O bonding are twice as large as the corresponding contributions of U-Cl bonding in complex **(d)**.

U-O bonding in **(e)** exhibits an *H*(**r**$_b$) value of $-$0.034 Ha/Å$^3$ related to a BSO *n* value of 0.268. Incorporation of S in the diamide ligand realized in complex in **(f)** enhances both the covalency of U-O and the corresponding bond strength, *H*(**r**$_b$) $=$ $-$0.078 Ha/Å$^3$ and BSO *n* $=$ 0.461. According to the QTAIM analysis, there is no bond critical point between sulfur and uranium, supporting the absence of any U-S bond. Despite the observed difference in the U-O bond strength in **(e)** and **(g)**, these complexes exhibit comparable U-O covalent character, despite the tridentate ligation found for complex **(g)**, which was confirmed by the existence of a bond critical point along U-N bond path, i.e., nitrogen is capable of binding uranium, in analogy with U-N bonding found in complexes **(b)** and **(c)**. Overall, U-N bonds in **(g)** are weaker and less covalent than the corresponding U-O bonds, following the trends of U-N bonds in **(b)** and **(c)**, and previous findings reported elsewhere [40].

As reported in previous work [6], uranium oxygen bonds in uranyl are stronger and more covalent than in neutral UO$_2$ in the $^3\Phi _u$ state, see Table 1. As stated above, the covalent character of U-O$_{ax}$ decreases as ligands are included in the uranyl moiety, qualitatively following the weakening of the axial bonding. Henceforth, our results are in partial support of Tsushima [134] explanation of the weakening of uranyl bonding upon complexation. One has to consider that the U-O$_{ax}$ bonds in complex **(f)** are slightly more covalent compared to the U-O$_{ax}$ in bonds**(b)**–**(d)**, despite having smaller local force constants, i.e., they are slightly weaker. On the other hand, Di Pietro and Kerridge [135] related the strength of U-O$_{ax}$ bonds of the uranyl moiety to the covalency of the uranium ligand bonds. In light of that, we correlated in Fig. 5 the overall equatorial covalency calculated from the sum of the *H*(**r**$_b$) values of all U-O, U-N, and U-Cl bonds with that of the corresponding U-O$_{ax}$ bonds strength. From this plot, we see that there is a qualitative trend that weaker U-O$_{ax}$ bonds are related to more covalent equatorial uranium-ligand bonds.

### Assessment of charge donations

We used the NBO analysis to calculate 2$^{nd}$-order stabilization energies $\Delta E^{(2)}$ defined by the donation of charge from a binding atom’s lone pairs into empty uranium orbitals. Figure 6a shows the relationship between $\Delta E^{(2)}$ and the local force constants of the corresponding equatorial uranium-ligand bonds, while Fig. 6b shows the relationship between the $\Delta E^{(2)}$ and the corresponding *H*(**r**$_b$) value. As revealed in Fig. 6, $\Delta E^{(2)}$ tends to increase with increasing strength and covalent character of the U-O and U-N ligand bonds. Following LMA and QTAIM results, $\Delta E^{(2)}$ tends to be smaller for nitrogen lone pair donations than for oxygen lone pair donations in line with increasing electronegativity.

The 2$^{nd}$-order perturbational analysis identified in complex **(a)** charge donations originating from both nitrate nitrogens and H$_2$O oxygens. Nonetheless, the corresponding stabilization energies are considerably smaller, measuring 10.51 and 2.70 kcal/mol for U-O$_{NO_3}$ and U-O$_{H_2O}$, respectively. In uranyl nitrate decorated with picolinamide-based ligands, i.e., complexes **(b)** and **(c)**, the 2$^{nd}$-order perturbational analysis identified donations from O$_{NO_3}$, O$_{^iPr}$, O$_{^tBu}$ and N, in line with previous assumptions that the picolinamide-based ligands have bidentate character. The magnitude of $\Delta E^{(2)}$ associated with charge donations originating from O$_{NO_3}$ is 104.5 kcal/mol on the average in line with their strength and covalency, see Table 1. Similarly, for U-O$_{^iPr}$, U-O$_{^tBu}$, and U-N bonds being weaker and less covalent, smaller values of $\Delta E^{(2)}$ are observed.

For complex **(d)**, the stabilization energy resulting from oxygen charge donations has an average value of 107.435 kcal/mol. Donations originating from the Cl atoms have corresponding $\Delta E^{(2)}$ values of 177.68 and 181.46 kcal/mol, thus reflecting the higher charge donation capability of Cl [43] in line with larger $k^a$ values and more negative *H*(**r**$_b$) values.

The glutaramide ligand provides the smallest stabilization energy of 6.89 kcal/mol. In contrast, oxygen charge donation in **(f)** is much more pronounced, with $\Delta E^{(2)}$ equal to 118 kcal/mol, correlating with the higher covalency and bond strength exhibited by the complex and corroborating the above assumption that the incorporation of S into the ligand alters the overall electronic structure. This result also confirms the previous hypothesis [36] that the geometry of **(f)** would favor charge donations from the oxygen towards uranium. We did not observe any charge donations from sulfur toward uranium, reinforcing the lack of interactions between those atoms. For **(g)**, we observed charge donations from (i) binding oxygens (with an average value of 98.9 kcal/mol) and (ii) the pyridine nitrogen, measuring 45.28 kcal/mol. The higher stabilization energy associated with oxygen donations in **(g)** correlates with the higher $k^a$ measured for the same interaction when compared to U-O in **(e)**. This result can explain why the U-O bonds in **(g)** are stronger than in **(e)**, despite the comparable degree of covalency in both complexes. Our $\Delta E^{(2)}$ results for complex **(g)** agree with a study conducted by Chen et al. [36], where NBO charges on uranium were addressed, and Dobler et al. [46] probes on La and Lu complexes featuring the same ligand, supporting the hypothesis that the nitrogen in the pyridine-substituted ligands effectively complexates uranium.

Following the suggestion that charge donations from the lone pairs of the uranium-ligand atoms into empty uranium orbitals are related to a weakening of the U-O$_{ax}$ bonds [44, 119, 136, 137], we correlated in Fig. 7 the overall $\Delta E^{(2)}$ contributions (i.e., the sum of all $\Delta E^{(2)}$ values for a given complex) with the corresponding $k^a$ (U-O$_{ax}$) values. From this plot, we observe that the overall charge donations are related to stronger U-O$_{ax}$ bonds for complexes **(a)** and **(e)**. However, there is no such relationship between $\Delta E^{(2)}$ and $k^a$ for the other complexes. Complexes **(b)**–**(d)**,**(f)** exhibit U-O$_{ax}$ local force constants below 7.5 mdyn/Å, while the overall $\Delta E^{(2)}$ contributions in those systems are above 470 kcal/mol. Complex **(e)** has stronger U-O$_{ax}$ interactions, despite its higher overall stabilization energy (472 kcal/mol), putting a question mark on this suggestion.

## Conclusions

We investigated in this work a series of uranyl and uranyl nitrate chelates, focusing on assessing equatorial uranium-ligand bonding including the question of bidentate versus tridentate ligation, and we probed the change of the U-O$_{ax}$ bond strengths as ligands are incorporated into the uranyl moiety. LMA paired with QTAIM and NBO analyses were applied leading to the following interesting results:

- Picolinamide-based ligands bind uranyl in a bidentate fashion, a result that is supported by both LMA, QTAIM, and NBO analyses. Interesting to note is that bulky picolinamide substituents as realized in complexes **(b)** and **(c)** do not influence the picolinamide-uranium ligand binding.
- In complex **(d)**, the Schiff base operates as a bidentate ligand, where binding occurs through oxygen atoms. The resulting U-O bonds are stronger and more covalent than the majority of U-O bonds in the other complexes studied. The Schiff base ligand is further stabilized by an intramolecular hydrogen bonding.
- Incorporation of sulfur in diamide ligands as realized in complex **(f)** considerably increases the strength of U-O bonds; nonetheless, no U-S bond was found, obliterating previous speculations in the literature. Pyridine-2,6-dicarboxamide ligands (see complex**(g)**) enhance the strength of U-O interactions to a smaller extent, although they provide an extra binding site via the nitrogen atom of the pyridine, which leads to a tridentate chelation. Interestingly, the increase of the U-O bond strength in complex **(g)** is predominantly a result of lone pair charge donations into empty uranium orbitals rather than the result of tridentate chelation.
- The strength of the U-O$_{ax}$ bonds decreases as ligands are incorporated into the uranyl moiety. These findings are better explained by the overall covalency of the equatorial interactions than via charge donations effects suggested in the literature.
- In summary, our results suggest pyridine- and S-substituted diamides as highly efficient uranyl extractors.

We hope the data obtained from our study will help fine-tuning new uranyl extractants.

### Supplementary information

Additional information is provided in the Supporting Information which contains cartesian coordinates of UO$_2^{2+}$ and molecules **(a)**-**(g)**.

## Supplementary Information

Below is the link to the electronic supplementary material.

Supplementary file 1 (pdf 87 KB)

## Data Availability

All research data supporting the results if contained in the tables and figures and text of the manuscript as well as in the Supporting information.
