# Supplementary material for: Extraction of uranyl from spent nuclear fuel wastewater via complexation—a local vibrational mode study
Source: J Mol Model. 2024 Jun 18;30(7):216. doi: 10.1007/s00894-024-06000-4 (PMC11614994; doi:10.1007/s00894-024-06000-4)
Supplement: Supplementary file 1 — (pdf 87 KB) [file 894_2024_6000_MOESM1_ESM.pdf]

Extraction of uranyl from spent nuclear fuel wastewater via complexation - a local vibrational mode study
SUPPORTING INFORMATION

Bárbara M. T. C. Peluzo[1], Renaldo T. Moura Jr.[1,2], Elfi Kraka[1*]

[1]Computational and Theoretical Chemistry Group (CATCO), Department of Chemistry, Southern Methodist University, 3215 Daniel Avenue, Dallas, 75275-0314, Texas, United States.
[2]Department of Chemistry and Physics, Center of Agrarian Sciences, Federal University of Paraíba, Areia, 58397-000, Paraíba, Brazil.

*Corresponding author(s). E-mail(s): ekraka@smu.edu;
Contributing authors: bmpeluzo@smu.edu, bmpeluzo.smu@gmail.com; renaldo.mourajr@cca.ufpb.br;

1 Optimized Coordinates

1.1 UO_2^{2+}

U 0.000000 0.000000 0.000000
O 0.000000 0.000000 1.681700
O 0.000000 0.000000 -1.681700

1.2 (a)

U 0.000000 0.000000 0.000000
O 0.000000 1.065847 2.214806
O -1.746884 0.051235 -0.000000
O 1.746884 -0.051235 -0.000000
N -0.000000 -0.000000 2.908257
O -0.000000 -1.065847 2.214806
O -0.000000 -0.000000 4.097161
O 0.000000 1.065847 -2.214806
O -0.000000 -1.065847 -2.214806
N -0.000000 -0.000000 -2.908257
O -0.000000 -0.000000 -4.097161
O 0.046110 2.527021 -0.000000
H -0.302511 2.967230 -0.779616
H -0.302511 2.967230 0.779616
O -0.046110 -2.527021 -0.000000
H 0.302511 -2.967230 -0.779616
H 0.302511 -2.967230 0.779616

1.3 (b)

U -1.276604 -0.131304 -0.113136
O -2.153677 2.000122 0.739903
O -2.786035 -2.048600 -0.029395
O -1.819773 0.290007 -1.722814
O -0.659825 -0.487235 1.492209
N -3.325047 1.586079 1.039963
N -1.986559 -2.912810 -0.499934
O -3.514095 0.362767 0.803614
O -0.839804 -2.450733 -0.798059
N 0.508113 1.935849 -0.268806
O 1.010060 -0.532922 -0.888982
C 1.976629 0.105125 -0.435290
C 1.788071 1.595775 -0.462911
C 4.091022 0.214203 0.800283
C 5.425611 0.386187 0.081813
C 4.275979 -0.363630 2.196750
H 3.668959 1.205910 0.947376
H 5.292381 0.671191 -0.962906
H 5.998612 1.171445 0.578721
H 6.026726 -0.520606 0.103544
H 3.325616 -0.442464 2.723897
H 4.751200 -1.343511 2.183735
H 4.924294 0.306822 2.763612
N 3.075300 -0.507849 0.010409
C 3.072280 -1.988394 -0.157830
C 2.374438 -2.705782 0.990894
C 4.441746 -2.570498 -0.456584
H 2.454980 -2.141607 -1.041309
H 1.413551 -2.246060 1.217799
H 2.180683 -3.736843 0.691316
H 2.980701 -2.728175 1.896038
H 4.918174 -2.083725 -1.307745
H 5.119444 -2.535988 0.396657
H 4.303208 -3.621830 -0.712672
C 2.754471 2.523642 -0.813108
C 2.387683 3.854604 -0.939607
H 3.120198 4.596572 -1.231752
C 1.077711 4.210631 -0.691614
H 0.744961 5.236666 -0.770508
C 0.168634 3.217062 -0.357972
H -0.870316 3.447434 -0.163522
H 3.769763 2.214300 -1.012460
O -2.280236 -4.059480 -0.648460

O -4.154598 2.307070 1.503025

1.4 (c)

U -1.450785 -0.195947 -0.083579
O -2.491186 1.956556 0.481605
O -2.828583 -2.193002 0.198325
O -1.970066 -0.007150 -1.744114
O -0.856198 -0.311912 1.564627
N -3.638609 1.498878 0.808245
N -1.962470 -3.045672 -0.161303
O -3.735916 0.244716 0.718037
O -0.843630 -2.538485 -0.494281
N 0.190309 1.929317 -0.435526
O 0.911227 -0.562569 -0.753335
C 1.806825 0.190788 -0.349952
C 1.492244 1.643477 -0.528788
N 3.017046 -0.245044 0.065768
C 2.431999 2.562524 -0.960428
C 2.011408 3.846036 -1.269668
H 2.718767 4.584998 -1.624884
C 0.674232 4.159435 -1.121048
H 0.297544 5.148526 -1.343692
C -0.204557 3.169714 -0.703305
H -1.261441 3.368982 -0.586610
H 3.463866 2.262532 -1.070221
O -2.172329 -4.219125 -0.185357
O -4.528438 2.207335 1.165426
C 3.499498 -1.528584 -0.591237
C 5.022984 -1.634762 -0.512742
C 2.849963 -2.791103 -0.022021
C 3.188680 -1.450175 -2.093528
H 5.507322 -0.793206 -1.009828
H 5.417249 -1.722031 0.495613
H 5.310788 -2.541584 -1.045372
H 1.764528 -2.731772 -0.066631
H 3.162280 -3.642483 -0.630459
H 3.152784 -2.994963 1.002474
H 3.729633 -2.252923 -2.594660
H 2.133500 -1.574091 -2.316358
H 3.536022 -0.503919 -2.515078
C 3.571952 0.286746 1.375056
C 4.911591 1.002724 1.209096
C 2.599539 1.264217 2.049284
C 3.703241 -0.864981 2.379669
H 5.708681 0.348775 0.868439
H 4.830958 1.838280 0.513599
H 5.211233 1.412517 2.175424

H 1.587183 0.860377 2.118870
H 2.954957 1.420576 3.068019
H 2.563900 2.241208 1.574714
H 4.069656 -0.449838 3.319657
H 2.732602 -1.322929 2.570802
H 4.399645 -1.639503 2.075417

1.5 (d)

U -0.009864 -1.008177 -1.164076
O -0.014241 -0.066624 -2.644028
O -1.549640 0.626182 -0.285433
O -0.008336 -1.812074 0.399780
O 1.537616 0.618447 -0.300374
N -1.438264 3.187036 -0.013712
H -1.087928 2.257103 -0.299961
N 1.335350 3.181918 0.005367
H 1.023826 2.257152 -0.332849
C 2.623164 0.627475 0.403856
C -2.679307 0.617841 0.346916
C -0.752682 4.372259 -0.275790
C 0.651987 4.369641 -0.267022
C 3.347148 -0.528972 0.669325
H 2.979560 -1.437184 0.215390
C 2.463823 3.081737 0.695322
H 2.864122 3.999629 1.115354
C 4.510845 -0.520828 1.445644
N 5.179942 -1.662747 1.721218
C 3.116552 1.886172 0.923307
C -2.608345 3.078349 0.603337
H -2.940118 -1.466039 0.220650
C 4.983925 0.735099 1.958371
H 5.886522 0.783362 2.546979
C 1.337061 5.555765 -0.501372
H 2.419114 5.539813 -0.537434
C 4.304362 1.875854 1.698596
H 4.673459 2.818336 2.089511
C 4.716948 -2.948499 1.211257
H 5.063957 -3.709422 1.912916
H 3.627445 -2.969798 1.250138
C 6.418259 -1.657609 2.481748
H 6.305411 -1.006635 3.351682
H 6.548147 -2.665059 2.878709
C 5.193511 -3.277605 -0.193910
H 6.281485 -3.348666 -0.240018
H 4.856193 -2.529187 -0.910548
C 7.653124 -1.263007 1.684398
H 8.532773 -1.267623 2.331572
H 7.831838 -1.958732 0.864635
H 7.549283 -0.264563 1.257820
Cl -2.020358 -2.509041 -1.981368
Cl 2.000454 -2.494895 -1.987955
C -3.242168 1.869626 0.812204

C -3.379466 -0.554026 0.594640
C -1.428088 5.562351 -0.521563
H -3.058071 3.993715 0.975862
C 0.652429 6.739174 -0.713554
H 4.774120 -4.234518 -0.507611
C -4.476683 1.834192 1.512110
C -4.604101 -0.566698 1.268171
H -2.509438 5.554424 -0.573997
C -0.733792 6.741609 -0.724147
H 1.203095 7.652105 -0.899197
H -4.898368 2.771420 1.860139
C -5.141221 0.678053 1.742840
N -5.274598 -1.721244 1.459386
H -1.277658 7.656943 -0.918244
H -6.089528 0.706489 2.257401
C -6.489194 -1.800973 2.248840
C -4.810421 -2.975556 0.878888
H -6.431528 -1.099634 3.082094
C -7.755000 -1.573015 1.437969
H -6.511853 -2.795992 2.699007
C -3.822043 -3.720157 1.760418
H -5.696781 -3.590558 0.706874
H -4.374498 -2.782779 -0.102094
H -8.637940 -1.682664 2.070785
H -7.831433 -2.295138 0.623310
H -7.767357 -0.574002 0.999837
H -2.915105 -3.135535 1.915969
H -4.256073 -3.951410 2.735887
H -3.534798 -4.657822 1.282706

1.6 (e)

C -3.704414 0.190799 1.327454
H -4.237082 1.132664 1.481407
H -4.435101 -0.615215 1.423808
C -2.991411 0.154307 0.000000
H -2.316051 1.017535 0.000000
H -2.426787 -0.785560 0.000000
C -3.704414 0.190799 -1.327454
H -4.237082 1.132664 -1.481407
H -4.435101 -0.615215 -1.423808
C -2.614815 0.030386 2.374048
O -1.413399 -0.003019 1.999443
N -2.938483 -0.092149 3.647535
C -2.614815 0.030386 -2.374048
O -1.413399 -0.003019 -1.999443
N -2.938483 -0.092149 -3.647535
C -1.918385 -0.325188 -4.654773
H -1.001753 -0.650011 -4.173998
H -2.269582 -1.098814 -5.337763
H -1.735540 0.587683 -5.225741
C -4.297441 -0.013102 -4.154428
H -4.620102 -0.990735 -4.517752
H -4.989946 0.330355 -3.393581
H -4.325885 0.691738 -4.986324
C -4.297441 -0.013102 4.154428
H -4.989946 0.330355 3.393581
H -4.620102 -0.990735 4.517752
H -4.325885 0.691738 4.986324
C -1.918385 -0.325188 4.654773
H -1.001753 -0.650011 4.173998
H -1.735540 0.587683 5.225741
H -2.269582 -1.098814 5.337763
C 3.704414 -0.190799 1.327454
H 4.237082 -1.132664 1.481407
H 4.435101 0.615215 1.423808
C 2.991411 -0.154307 0.000000
H 2.316051 -1.017535 0.000000
H 2.426787 0.785560 0.000000
C 3.704414 -0.190799 -1.327454
H 4.237082 -1.132664 -1.481407
H 4.435101 0.615215 -1.423808
C 2.614815 -0.030386 2.374048
O 1.413399 0.003019 1.999443
N 2.938483 0.092149 3.647535
C 2.614815 -0.030386 -2.374048

O 1.413399 0.003019 -1.999443
N 2.938483 0.092149 -3.647535
C 1.918385 0.325188 -4.654773
H 1.001753 0.650011 -4.173998
H 2.269582 1.098814 -5.337763
H 1.735540 -0.587683 -5.225741
C 4.297441 0.013102 -4.154428
H 4.620102 0.990735 -4.517752
H 4.989946 -0.330355 -3.393581
H 4.325885 -0.691738 -4.986324
C 4.297441 0.013102 4.154428
H 4.989946 -0.330355 3.393581
H 4.620102 0.990735 4.517752
H 4.325885 -0.691738 4.986324
C 1.918385 0.325188 4.654773
H 1.001753 0.650011 4.173998
H 1.735540 -0.587683 5.225741
H 2.269582 1.098814 5.337763
U 0.000000 0.000000 0.000000
O -0.046849 -1.740455 0.000000
O 0.046849 1.740455 0.000000

1.7 (f)

C 2.863507 1.888956 0.451216
O 1.730777 1.562193 -0.006573
N 3.341154 3.105287 0.297709
C 4.658773 3.499895 0.787317
C 2.576180 4.137355 -0.384890
H 4.925220 4.442287 0.315624
H 4.650447 3.648207 1.868410
H 5.412976 2.763856 0.514775
H 1.604841 3.749611 -0.670656
H 2.453015 4.993090 0.280135
H 3.115732 4.460158 -1.276385
C 3.677925 0.837937 1.147314
H 3.005817 0.125016 1.621657
H 4.328804 1.253162 1.914862
S 4.813585 -0.000000 -0.000000
C 2.863507 -1.888956 -0.451216
O 1.730777 -1.562193 0.006573
N 3.341154 -3.105287 -0.297709
C 4.658773 -3.499895 -0.787317
C 2.576180 -4.137355 0.384890
H 4.925220 -4.442287 -0.315624
H 4.650447 -3.648207 -1.868410
H 5.412976 -2.763856 -0.514775
H 1.604841 -3.749611 0.670656
H 2.453015 -4.993090 -0.280135
H 3.115732 -4.460158 1.276385
C -2.863507 -1.888956 0.451216
O -1.730777 -1.562193 -0.006573
N -3.341154 -3.105287 0.297709
C -4.658773 -3.499895 0.787317
C -2.576180 -4.137355 -0.384890
H -4.925220 -4.442287 0.315624
H -4.650447 -3.648207 1.868410
H -5.412976 -2.763856 0.514775
H -1.604841 -3.749611 -0.670656
H -2.453015 -4.993090 0.280135
H -3.115732 -4.460158 -1.276385
C -3.677925 -0.837937 1.147314
H -3.005817 -0.125016 1.621657
H -4.328804 -1.253162 1.914862
S -4.813585 0.000000 -0.000000
C -2.863507 1.888956 -0.451216
O -1.730777 1.562193 0.006573
N -3.341154 3.105287 -0.297709

C -4.658773 3.499895 -0.787317
C -2.576180 4.137355 0.384890
H -4.925220 4.442287 -0.315624
H -4.650447 3.648207 -1.868410
H -5.412976 2.763856 -0.514775
H -1.604841 3.749611 0.670656
H -2.453015 4.993090 -0.280135
H -3.115732 4.460158 1.276385
C 3.677925 -0.837937 -1.147314
H 3.005817 -0.125016 -1.621657
H 4.328804 -1.253162 -1.914862
C -3.677925 0.837937 -1.147314
H -3.005817 0.125016 -1.621657
H -4.328804 1.253162 -1.914862
U 0.000000 0.000000 0.000000
O 0.000000 0.000000 1.747795
O 0.000000 -0.000000 -1.747795

1.8 (g)

U 0.000000 0.000000 0.004984
O -0.000000 1.742098 0.005945
O -0.000000 -1.742098 0.005945
C -3.348341 0.562413 1.103158
C -4.530222 1.290317 1.083288
C -5.112098 1.569805 -0.141256
C -4.535346 1.069456 -1.295868
C -3.348689 0.358660 -1.184707
N -2.744437 0.169609 -0.014988
H -6.009384 2.172930 -0.195077
H -4.967597 1.672289 1.993509
H -4.977659 1.272042 -2.260111
C -2.504928 -0.157500 -2.306460
O -1.277971 -0.144703 -2.094819
C -2.516312 0.237796 2.302732
O -1.295249 0.110413 2.095053
N -3.021698 -0.600900 -3.442672
N -3.034442 0.102815 3.515139
C -4.431307 -0.855648 -3.689953
H -4.869124 -0.078495 -4.320102
H -4.515459 -1.806265 -4.216769
H -4.988267 -0.939537 -2.761833
C -4.444609 -0.070558 3.824426
H -5.011958 -0.353742 2.943191
H -4.533704 -0.879090 4.550383
H -4.867363 0.833158 4.268437
C -2.132787 -1.001405 -4.522271
H -2.138254 -2.088260 -4.625421
H -2.485989 -0.558178 -5.454070
H -1.124504 -0.665698 -4.306873
C -2.133388 -0.062974 4.644911
H -2.571728 0.430086 5.511927
H -1.994489 -1.122396 4.872291
H -1.173161 0.385708 4.411482
C 3.348341 -0.562413 1.103158
C 4.530222 -1.290317 1.083288
C 5.112098 -1.569805 -0.141256
C 4.535346 -1.069456 -1.295868
C 3.348689 -0.358660 -1.184707
N 2.744437 -0.169609 -0.014988
H 6.009384 -2.172930 -0.195077
H 4.967597 -1.672289 1.993509
H 4.977659 -1.272042 -2.260111
C 2.504928 0.157500 -2.306460

O 1.277971 0.144703 -2.094819
C 2.516312 -0.237796 2.302732
O 1.295249 -0.110413 2.095053
N 3.021698 0.600900 -3.442672
N 3.034442 -0.102815 3.515139
C 4.431307 0.855648 -3.689953
H 4.869124 0.078495 -4.320102
H 4.515459 1.806265 -4.216769
H 4.988267 0.939537 -2.761833
C 4.444609 0.070558 3.824426
H 5.011958 0.353742 2.943191
H 4.533704 0.879090 4.550383
H 4.867363 -0.833158 4.268437
C 2.132787 1.001405 -4.522271
H 2.138254 2.088260 -4.625421
H 2.485989 0.558178 -5.454070
H 1.124504 0.665698 -4.306873
C 2.133388 0.062974 4.644911
H 2.571728 -0.430086 5.511927
H 1.994489 1.122396 4.872291
H 1.173161 -0.385708 4.411482
